# Development of the Horse Grimace Scale (HGS) as a Pain Assessment Tool in Horses Undergoing Routine Castration

**DOI:** 10.1371/journal.pone.0092281

**Published:** 2014-03-19

**Authors:** Emanuela Dalla Costa, Michela Minero, Dirk Lebelt, Diana Stucke, Elisabetta Canali, Matthew C. Leach

**Affiliations:** 1 Università degli Studi di Milano, Dipartimento di Scienze Veterinarie e Sanità Pubblica, Milan, Italy; 2 Pferdeklinik Havelland / Havelland Equine Hospital, Beetzsee-Brielow, Germany; 3 Newcastle University, School of Agriculture, Food & Rural Development, Newcastle upon Tyne, United Kingdom; ETH Zurich, Switzerland

## Abstract

**Background:**

The assessment of pain is critical for the welfare of horses, in particular when pain is induced by common management procedures such as castration. Existing pain assessment methods have several limitations, which reduce the applicability in everyday life. Assessment of facial expression changes, as a novel means of pain scoring, may offer numerous advantages and overcome some of these limitations. The objective of this study was to develop and validate a standardised pain scale based on facial expressions in horses (Horse Grimace Scale [HGS]).

**Methodology/Principal Findings:**

Forty stallions were assigned to one of two treatments and all animals underwent routine surgical castration under general anaesthesia. Group A (n = 19) received a single injection of Flunixin immediately before anaesthesia. Group B (n = 21) received Flunixin immediately before anaesthesia and then again, as an oral administration, six hours after the surgery. In addition, six horses were used as anaesthesia controls (C). These animals underwent non-invasive, indolent procedures, received the same treatment as group A, but did not undergo surgical procedures that could be accompanied with surgical pain. Changes in behaviour, composite pain scale (CPS) scores and horse grimace scale (HGS) scores were assessed before and 8-hours post-procedure. Only horses undergoing castration (Groups A and B) showed significantly greater HGS and CPS scores at 8-hours post compared to pre operatively. Further, maintenance behaviours such as explorative behaviour and alertness were also reduced. No difference was observed between the two analgesic treatment groups.

**Conclusions:**

The Horse Grimace Scale potentially offers an effective and reliable method of assessing pain following routine castration in horses. However, auxiliary studies are required to evaluate different painful conditions and analgesic schedules.

## Introduction

The recognition and alleviation of pain is critical for the welfare of horses. Although considerable progress has been made in understanding physiology and treatment of pain in animals over the past 20 years, the assessment of pain in horses undergoing management procedures, such as branding, pin firing and castration, remains difficult and frequently suboptimal [1]–[4]. Equine castration is a husbandry practice routinely performed to: avoid undesired mating, facilitate handling, and reduce aggression and other undesirable behaviours. Annually, it is estimated that 240,000 horses are castrated in Europe [5]. Studies in other species demonstrate that animals experience pain and discomfort both acutely and chronically following castration [6], [7]. Despite the limited research in horses, castration has been shown to be associated with some degree of pain that can persist for several days and, therefore, requires adequate analgesic treatment [2]–[4], [8]. Price et al. [1] reported that only 36.9% of horses received analgesics for post operative pain, with one perioperative administration of Flunixin appearing to be one of the most common analgesic procedure provided following castration [9]: one possible explanation for this is the difficulty in assessing and quantifying pain in this species [2], [10]. For example, even though castration of horses is a common procedure, no gold standard for pain assessment is available to date. As in other animal species, pain in horses is difficult to assess because of their inability to communicate with humans in a meaningful manner. This could be further compounded by horses potentially suppressing the exhibition of obvious signs of pain in the presence of possible predators (i.e. humans) as is suggested with other prey species. Several behaviour-based assessments of pain in horses already exist [11]–[17]. The Post Abdominal Surgery Pain Assessment Scale (PASPAS) is a multidimensional scale that can be used to quantify pain after laparotomy [14]. The Composite Pain Scale (CPS) focuses on the presence of pain-related behaviours and the change in the frequency of normal behaviour patterns and physiological parameters [16]and has been successfully applied following both surgery (e.g. castration), injury and disease (e.g. laminitis, colic) [16], [17]. However, behaviour-based assessments of pain are not without limitations that constrain their routine application. These include the need for trained and experienced observers [8], [16], [17], prolonged observation periods [18], particularly in conditions inducing only mild pain, and the palpation of the painful area in some cases [14], [16], [17]. Furthermore, many of the pain related behaviours described so far have been identified in response to what are perceived to be severely painful conditions (e.g. colic, laminitis [14], [16]), rather than those that are perceived to be mildly to moderately painful conditions (e.g. identification procedures [19]). Recently, a new approach to pain assessment has been developed in rodents and rabbits utilising the assessment of facial expressions [20]–[23]. Facial expressions are commonly used to assess pain and other emotional states in humans, particularly in those who are unable to communicate coherently with their clinicians (e.g. those with cognitive impairment and neonates [24], [25]). In humans, facial expressions are routinely scored both manually [25] and automatically [26] using the Facial Action Coding System (FACS), which is considered as an accurate and reliable method that describes the changes to the surface appearance of the face resulting from individual or combinations of muscle actions, referred to as ‘action units’ [27]. Action units relating to pain have been identified in rodents and rabbits and incorporated into species-specific “grimace scales” [20]–[23]. These grimace scales are considered to give a number of advantages over other routinely used methods of assessing pain in animals. Firstly, grimace scales are less time consuming to carry out [20]–[23]. Secondly, observers can easily and rapidly be trained to use them [20]–[23]. Thirdly, grimace scales may utilise our potential tendency to focus on the face when scoring pain [28], [29]. Fourthly, they can be used to effectively assess a range of painful conditions, from mild to severe pain [20]. Finally, it can increase the safety of the observer when assessing pain in large animals, as grimace scales do not require the observer to approach the subject and palpate the painful area for the assessment. Therefore the Horse Grimace Scale (HGS) may offer an effective and practical method of identifying painful conditions and the efficacy of the methods we use to ameliorate pain in horses (i.e. analgesia administration). Furthermore, it can be applied in association with other behaviour-based methods to enhance the assessment of pain in horses and could be implemented in practice by owners and stable managers as an effective on farm early warning system.

The objectives of this study were to develop and validate a standardised pain scale based on facial expressions in horses (Horse Grimace Scale) using routine castration, and to investigate whether the HGS could be successfully implemented with minimal training, enabling the development of an on-farm pain assessment tool. Castration was considered a suitable model for the development of HGS because it is amongst the most common management procedures carried out in veterinary practice. In addition, utilising animals that are undergoing routine castration for husbandry reasons allows the researchers to avoid carrying out a surgical procedure solely for the evaluation of a method of assessing post-procedural pain.

## Materials and Methods

### Ethics statement

Castration is a routinely conducted husbandry procedure that was carried out in compliance with the European Communities Council Directive of 24 November 1986 (No. 86/609/EEC). This study was registered as an animal experiment at the Brandenburg State Veterinary Authority (V3-2347-A-42-1-2012). Horses involved in this study underwent routine veterinary procedures for health or husbandry purposes at the request of their owner on a voluntary basis. Consequently, no animals underwent anaesthesia or surgery or were directly used in order to record data for the purposes of this study. Verbal informed consent was gained from each participant prior to taking part in this research. Written consent was deemed unnecessary as no personal details of the participants were recorded. No animals received less than the standard analgesic regimen for the purposes of the study. This study employed a strict “rescue” analgesia policy: if any animal was deemed to be in greater than mild pain (assessed live by an independent veterinarian), then additional, pain relieving medication would immediately be administered and the animal removed from the study. The choice of medication and dosage would be based on the severity of pain identified thorough the clinical examination of the individual horse.

### Animals and Husbandry

**Figure 1:**
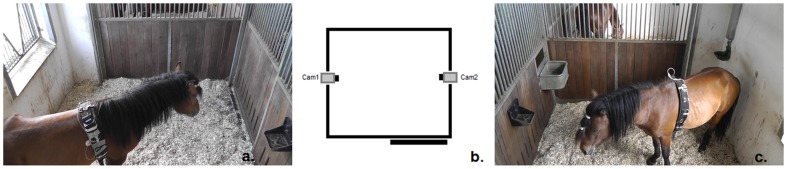
Video cameras position. The drawing in the middle (b) shows the position of the two HD cameras. Pictures on the left (a) and on the right (c) show frames grabbed from Cam1 and Cam2 respectively.

Forty stallions of different breeds, coat colour and aged between 1 and 5 years (mean age 2.3 years) underwent routine castration (see Table 1 for details). In addition, six horses of mixed age and gender that were undergoing general anaesthesia for different non-invasive and indolent procedures were used as a control group (see Table 2 for details). All animals were recruited from the hospital's clinical cases. In order to be included in this study, all the subjects had to be deemed healthy and without signs of cryptorchidism by an equine veterinarian after physical examination and behavioural evaluation. All horses were hospitalised in a veterinary clinic for 5 days to undergo castration or anaesthesia alone. In order to control for any possible effect of stress related to being in a novel environment and separated from their peers, all the subjects were allowed to acclimatise to their new environment, clinicians and video cameras for 2 days prior to the beginning of the study. In order to control for any possible differences in behaviour between stallions, geldings and mares, the acclimation period before starting with data collection was the same for all the horses. All subjects were kept in the same housing and management conditions: they were housed in standard single horse boxes (4×3 m with an outside window, see Figure 1) on wood shavings (German Horse Span Classic, German Horse Pellets, Wismar, Germany), and in visual contact with other conspecifics. They were fed twice a day with hay (approx. 3 kg/100 kg body weight per day) and water was provided ad libitum by automatic drinkers. Food was withheld from all horses for 8-hours before and 5 hours after anaesthesia (standard protocol for general anaesthesia [30]). In order to collect videos and images without disturbing the behaviour of the horses, two digital video cameras (Panasonic, HDC-SD99, Panasonic, Japan) were positioned on the top of the grate section on opposite sides of the box (see Figure 1).

**Table 1:** Breed and mean age of the stallions of the two treatment groups.

Group (N)	Breed (N)	Age (Mean)
Treatment A (19)	Arabian horse (1)	2
	German Warmblood (3)	2.6
	Friesian (3)	1.7
	Iceland pony (5)	2.6
	Irish draught horse (1)	2
	Polo horse (1)	2
	Quarter horse (3)	2
	Mini-Shetland pony (1)	2
	Tennessee Walker horse (1)	2
Treatment B (21)	German Warmblood (4)	2.5
	Edles Warmblood (1)	1
	Friesian (3)	1.7
	Iceland pony (6)	2.5
	Irish draught horse (1)	1
	Polo horse (2)	1.5
	Quarter horse (2)	2
	Mini-Shetland pony (1)	4
	Trakehner (1)	5

**Table 2:** Details of the horses of the control group.

Sex	Breed	Age	Procedure
Mare	Polo horse	7	control X-ray pelvis
Mare	German warmblood	14	control X-ray cervical
Gelding	Haflinger	3	hoof correction
Gelding	Haflinger	3	hoof correction
Gelding	Haflinger	4	teeth rasping
Gelding	Haflinger	2	hoof correction

### Surgery and Analgesic Treatment Groups

Horses undergoing castration were divided into two breed-matched treatment groups using a blocked randomization process. Group A (N = 19) received a single perioperative injection of Flunixin (1.1 mg/Kg i.v., Flunixin 5%, medistar, Aschberg, Germany) approximately 5 minutes prior to anaesthesia immediately after administration of sedative drug. Group B (N = 21) received a perioperative injection of Flunixin (1.1 mg/Kg i.v.) as for group A and a subsequent oral application of Flunixin (Flunidol 5%, cp-pharma, Burgdorf, Germany, 1.1 mg/Kg p.o.) 6 hours after castration. All the medications were administered by a veterinary nurse who was aware of group allocation; the veterinarians responsible for pain assessment were blinded to treatment group. Horses underwent routine surgery castration with closed technique through a scrotal approach without primary closure of the wound in dorsal recumbency under general anaesthesia [9], as recommended by the National Equine Welfare Council (NEWC) and the Canadian Veterinary Medical Association [31], [32]. The surgeries were all carried out by one of two equally experienced veterinary surgeons. To investigate the impact of general anaesthesia on the HGS, a control group (C) of horses was recruited. The control horses (N = 6) underwent the same general anaesthesia protocol as horses in groups A and B and received a single perioperative injection of Flunixin (1.1 mg/Kg i.v.) 5 minutes prior to anaesthesia. All castrated horses also received antibiotic treatment for three days starting at the morning before surgery (Synutrim 72% Pulver, Vétoquinol, Ravensburg, Germany), 2–4 mg Trimethoprim and 12 mg Sulfadiazin /Kg p.os every 12 h. Prior to the first drug application the weight of each horse was estimated with a weight tape in order for the correct drug doses to be administrated. The anaesthesia protocol was the same for all the subjects: pre-medication with Romifidine (Sedivet, Boehriger Ingelheim Vetmedica, Ingelheim, Germany, 80 micrograms Romifidinehydrochloride/Kg), induction with Diazepam (Diazepam-ratiopharm, Ratiopharm, Ulm, Germany, 0.1 mg/Kg) and Ketamine (Ketamin 10%, medistar, Ascheberg, Germany, 2.2 mg/Kg) intravenously via a jugular catheter. When necessary, general anaesthesia was maintained by another injection of Ketamine (1.1 mg/Kg). Twenty-six out of 40 castrated horses (65%) and 2 out of 6 control horses (33.3%) needed a second injection of Ketamine to maintain an appropriate level of anaesthesia in order to complete the surgery or the non-invasive procedure; the duration of anaesthesia was comparable long all the subjects. Surgery lasted 10–15 min, following which horses were moved to a recovery box; then, as soon as they were able to walk (20–60 minutes after anaesthesia), returned to their home box. Recovery from anaesthesia is the time that a horse need to stand up; it strongly depends on individual differences and it does not necessarily reflect the duration of previous anaesthesia. Horses recovered from anaesthesia without assistance inside the recovery box under visual supervision of a veterinary nurse. No intra-operative complications were reported and all horses recovered from anaesthesia fully and uneventfully prior to the first data collection post-procedure. All surgeries/general anaesthesia were carried out between 9 and 11am.

### Pain Assessment

At each time interval an overall pain assessment was conducted by two trained veterinarians blinded to treatment group using a Composite Pain Scale (CPS) (see Table S1) based on the one developed by Bussieres and colleagues [16], [17] and adapted according to Søndergaard and Halekoh [33].

### Video Recording

Thirty-minute video sequences were recorded using 2 High Definition Cameras with a 28 mm wide angle objective lens (Panasonic, HDC-SD99, Panasonic, Japan), the videos were recorded one day before procedure in the evening (baseline observation, pre-procedure) and at similar time 8-hours following procedure (8 h post-procedure). The cameras were positioned at opposite sides of the box, on the top of the grate section. This arrangement gave the highest probability of capturing the behaviour and face of the horse during filming without interfering with their normal behaviour (see Figure 1).

### Behavioural Recording

Behaviour of horses undergoing castration was evaluated. For each video, the last 15 minutes were analysed. A focal animal continuous recording method [34] was used to describe the horse's activity. The frequency and duration of thirty categories of behaviour (see Table S2) was continuously recorded using Solomon Coder (beta 12.09.04, copyright 2006–2008 by András Péter) by two trained treatment and session blind observers. Behaviours recorded as states (movement, licking and chewing, alertness, agitation, investigative behaviour, drinking, eating, lowered head carriage, head orientation, grooming) were reported as durations, and those recorded as events (weight-shifting, pawing, kicking, flank watching, rolling, yawning, masturbating, vocalization, urinating, defecating, tail swishing, flehmen) were reported as frequency of occurrence. Duration of maintenance behaviours showing the same pattern were added to form the composite maintenance behaviour score, comprising exploration, alertness and grooming.

### Horse Grimace Scale (HGS) Recording

**Figure 2:**
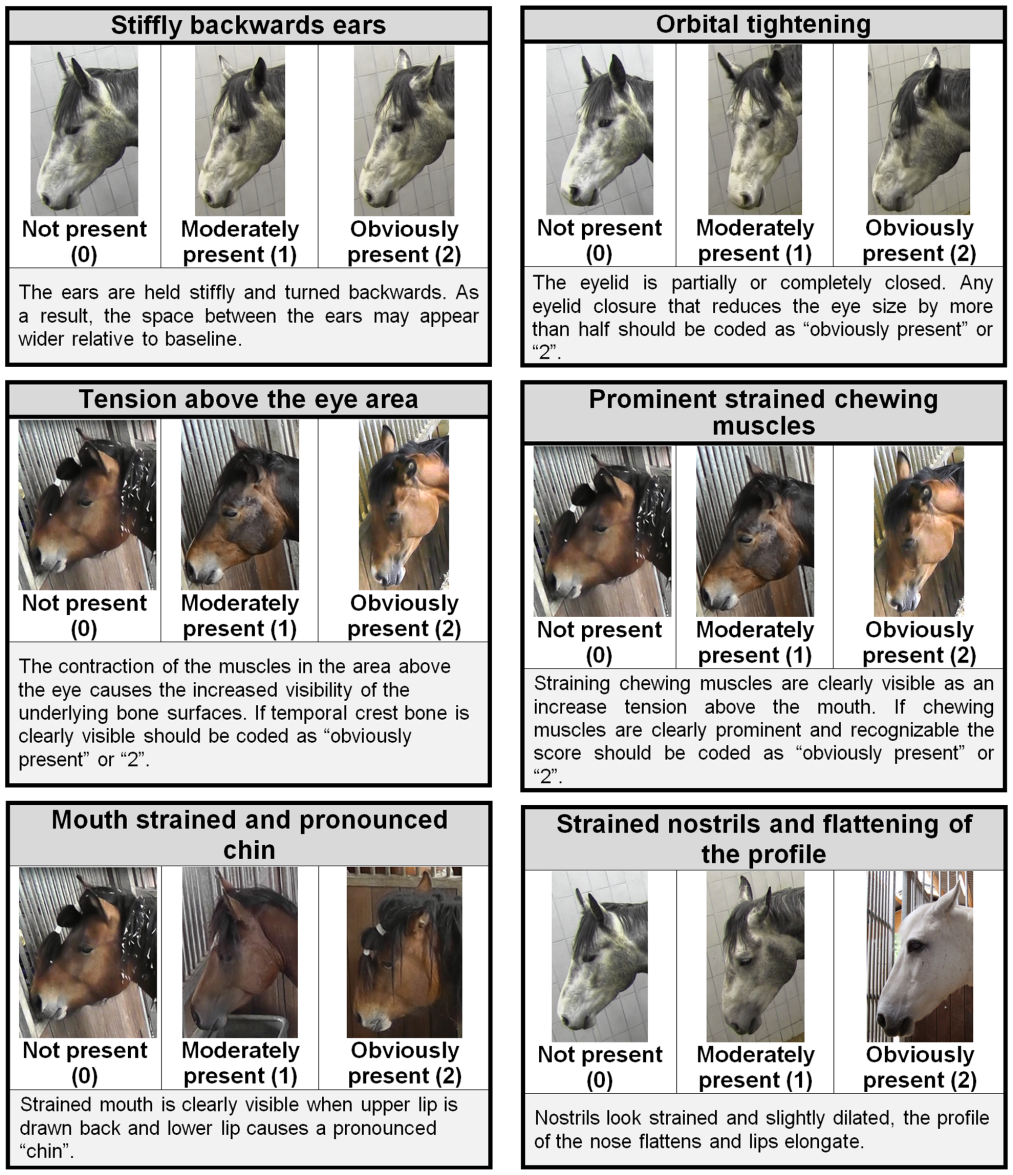
Horse Grimace Pain Scale (HGS). The Horse Grimace Pain Scale with images and explanations for each of the 6 facial action units (FAUs). Each FAU is scored according to whether it is not present (score of 0), moderately present (score of 1) and obliviously present (score of 2).

The HGS was created following the methods developed by Langford et al. [20] and Sotocinal et al. [21] for rodents and Keating et al. [23] for rabbits. Changes in horse behaviour and facial expressions were identified using a pilot study [8] following eight stallions undergoing surgical castration with the same anaesthetic and analgesic protocol as used in the main study. According to the published literature [2], [4] and pilot study results [8], 8-hours post-castration was deemed the appropriate time interval between observations as this was when the most of the pain related behaviours were observed. Furthermore, the estimated duration of sedation from pre-medication drugs and anaesthetics used in this study should have subsided at 8-hours post-intervention [35]–[37]. Still images were extracted from each video sequences whenever the horse was found in a position with the head and face clearly visible. This enabled a number of clear and high quality images to be extracted. Each image was then cropped so that only the head of the horse was visible to prevent observers from being biased by the body of the animal when looking at each image. Images of each subject before and 8-hours after surgery were compared to identify changes in facial expressions associated with these procedures by a trained treatment blind observer experienced in assessing facial expressions in other species (MCL). Based on these comparisons, the Horse Grimace Scale (HGS) was developed, and comprises six facial action units (FAUs): stiffly backwards ears, orbital tightening, tension above the eye area, prominent strained chewing muscles, mouth strained and pronounced chin, strained nostrils and flattening of the profile (see Figure 2). One hundred and twenty six images were randomly selected by a non-participating assistant with no experience of assessing pain in horses for further scoring (63 pre and 63 post procedure images). In order to maintain a balanced design for the statistical analysis, the image set comprised 1 or 2 pictures of each horse pre and 8-hours post procedure (e.g. lateral images pre and post and frontal images pre and post). The 126 images were then scored in a random order using the Horse Grimace Scale by five treatment and session (pre or post-surgery) blind observers. A detailed hand out with the description of the six identified FAUs and the scoring system was distributed to the observers (see Figure 2). Briefly, for each image each observer was asked to give a score for each of FAU using a 3-point scale (0 =  not present, 1 =  moderately present, 2 =  obviously present). If the participant was unable to score a particular FAU clearly, they were asked to score it as ‘I don't know’. The Horse Grimace Scale (HGS) score was determined by adding the individual scores for each of the six action units identified (stiffly backwards ears, orbital tightening, tension above the eye area, prominent strained chewing muscles, mouth strained and pronounced chin and strained nostrils and flattening of the profile) in each image. Consequently, the maximum possible HGS score was 12 (i.e. a score of 2 for each of the 6 FAUs). In addition, the observers were asked to make a global pain judgment for each picture (no pain vs. pain) based upon their own clinical experience. If they deemed the individual to be in pain, then they were asked to score the intensity of that pain (mild, moderate or severe). In order to explore the effect of time (pre vs. post-procedure) and treatment (analgesia and surgery), the mean HGS scores were calculated for each image across all participants.

### Observer Selection

Five observers were selected as they had expertise either with horses or scoring facial expressions. The observers had diverse backgrounds including horse welfare researchers, veterinary surgeons, research scientists and veterinary students.

### Statistical Analysis

All statistical analyses were conducted using SPSS 19 (SPSS Inc., Chicago, USA). Differences were considered to be statistically significant if P≤0.05. The data were tested for normality and homogeneity of variance using Kolmogorov-Smirnov and Levene test, respectively. CPS and HGS scores were not normally distributed and therefore the scores were transformed using square root transformation. Repeated Measures General Linear Model (RGLM) was used to analyse the data with the time points (pre and 8-hours post-procedure) as the *within-subjects* factor and the treatment group as the *between-subjects* factor. Any treatment effects were further investigated using analysis of variance (ANOVA) with data from the separate time periods forming the dependent variables and treatment as the fixed effect. Post-hoc analysis of treatment group effects was conducted using Bonferroni post-hoc test. The reliability of HGS scale was determined using inter-class correlation coefficient (ICC) to compare mean scores for each of the facial action units across all the participants. Accuracy was determined by comparing the global pain and no pain judgement made by the treatment and period blind observers with actual pain state of the horse in each photograph. The reliability of the Composite Pain Scale scores were analysed using an inter-class correlation coefficient (ICC). Reliability of the manual behaviour analysis was assessed by means of independent parallel coding of a random sample of videotaped sessions (5 clips) using percentage agreement. Wilcoxon test was conducted to determine differences in behaviour shown before and 8 hour after procedure. Spearman correlation coefficients were calculated to investigate the relationship between the CPS, HGS and behaviour.

## Results

During this study, no horses required the administration of rescue analgesia or had to be removed from the study due to adverse events.

### Horse Grimace Scale (HGS)

**Figure 3:**
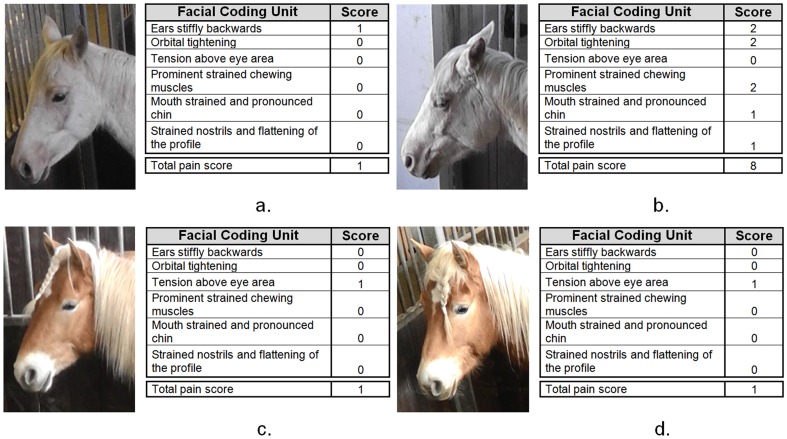
Mean Horse Grimace Scale (HGS) scores pre and 8-hours post-procedure. HGS scores are presented on the y-axis (±1 SE) for horses undergoing routine castration (A and B), and anaesthesia control group (C) with the pre and 8-hours post-procedure recordings on the x-axis (** P = 0.000).

**Figure 4:**
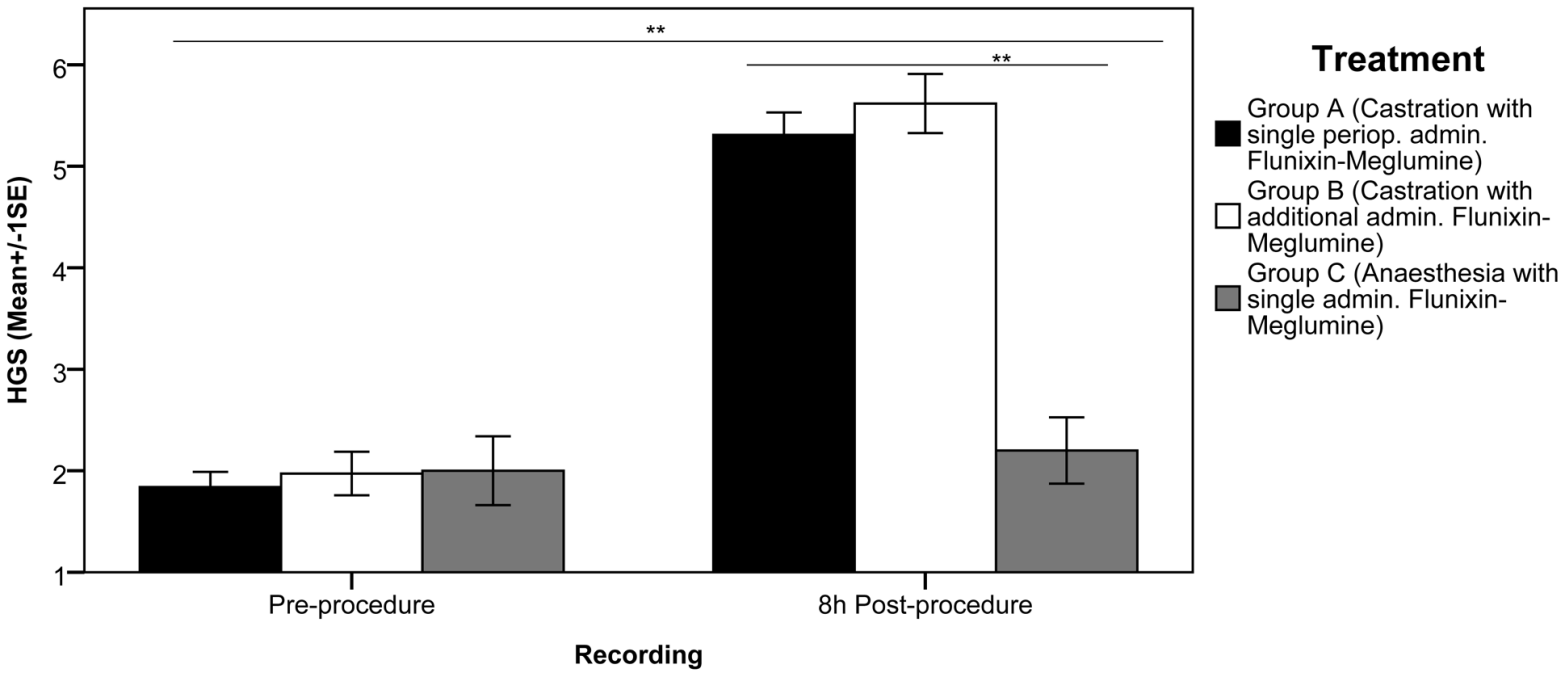
Example images and HGS scores. Example images and associated HGS scores of the same horse pre (a; c) and 8-hours post-procedure (b; d). Images a and b underwent castration; c and d were control animals.

Time, treatment and time*treatment interaction had significant effects on HGS score (RGLM, P = 0.000, P = 0.007 and P = 0.000, respectively; η^2^ = 0.03). In the pre-procedure period there was no significant difference between the three treatments (ANOVA, P = 0.84; η^2^ = 0.00). At eight-hours post-procedure the HGS score was significantly different between the three treatments (ANOVA, P = 0.000; η^2^ = 0.11), with the HGS score being significantly higher in horses undergoing routine castration (Groups A and B) compared to the control group (Group C) (Bonferroni post-hoc, P = 0.000 for both comparisons). No significant differences were found between groups with the single (A) or multiple (B) Flunixin administration (Bonferroni post-hoc, P = 1.000) (see Figure 3). Example images and associated HGS scores of horses in groups undergoing castration compared to control are shown in Figure 4.

Total observation time was approximately 40 minutes for scoring all the pictures. The average accuracy of global pain judgement was 73.3%, with false positives being slightly more prevalent (17.0%) than misses (false negatives) (9.8%). Individual accuracy of participants varied from 67.5% to 77.8%. The Horse Grimace Scale demonstrated high inter observer reliability with an overall Intraclass Correlation Coefficient (ICC) value of 0.92. The individual action units comprising the HGS also showed high ICC values of: 0.97 for stiffly backwards ears, 0.83 for orbital tightening, 0.86 for tension above the eye area, 0.88 for prominent strained chewing muscles, and 0.72 for mouth strained and pronounced chin. The only exception was for strained nostrils and flattening of the profile (ICC = 0.58). On average, all the six facial action units (FAUs) were assessed easily by all the participants, as shown by the percentage of “not able to score” ranging from 0% for ear position to 21% for the tension above eye and strained mouth and pronounced chin (see Table 3). Front-view images were more difficult to score than profile view images, in particular for the evaluation of prominent strained chewing muscles and mouth strained and pronounced chin (46% and 81% respectively of “not able to score”). In profile view images, horses with dark-brown or black coats were more difficult to score than grey and light brown coat, especially for the orbital tightening and prominent strained chewing muscles (12% and 16% respectively).

**Table 3:** The percentage of “not able to score” for each Facial Action Unit identified.

Facial Action Units (FAUs)	Not able to score (%)
Stiffly backwards ears	0
Orbital tightening	9
Tension above the eye area	21
Prominent strained chewing muscles	15
Mouth strained and pronounced chin	21
Strained nostrils and flattening of the profile	8

### Composite Pain Scale (CPS)

**Figure 5:**
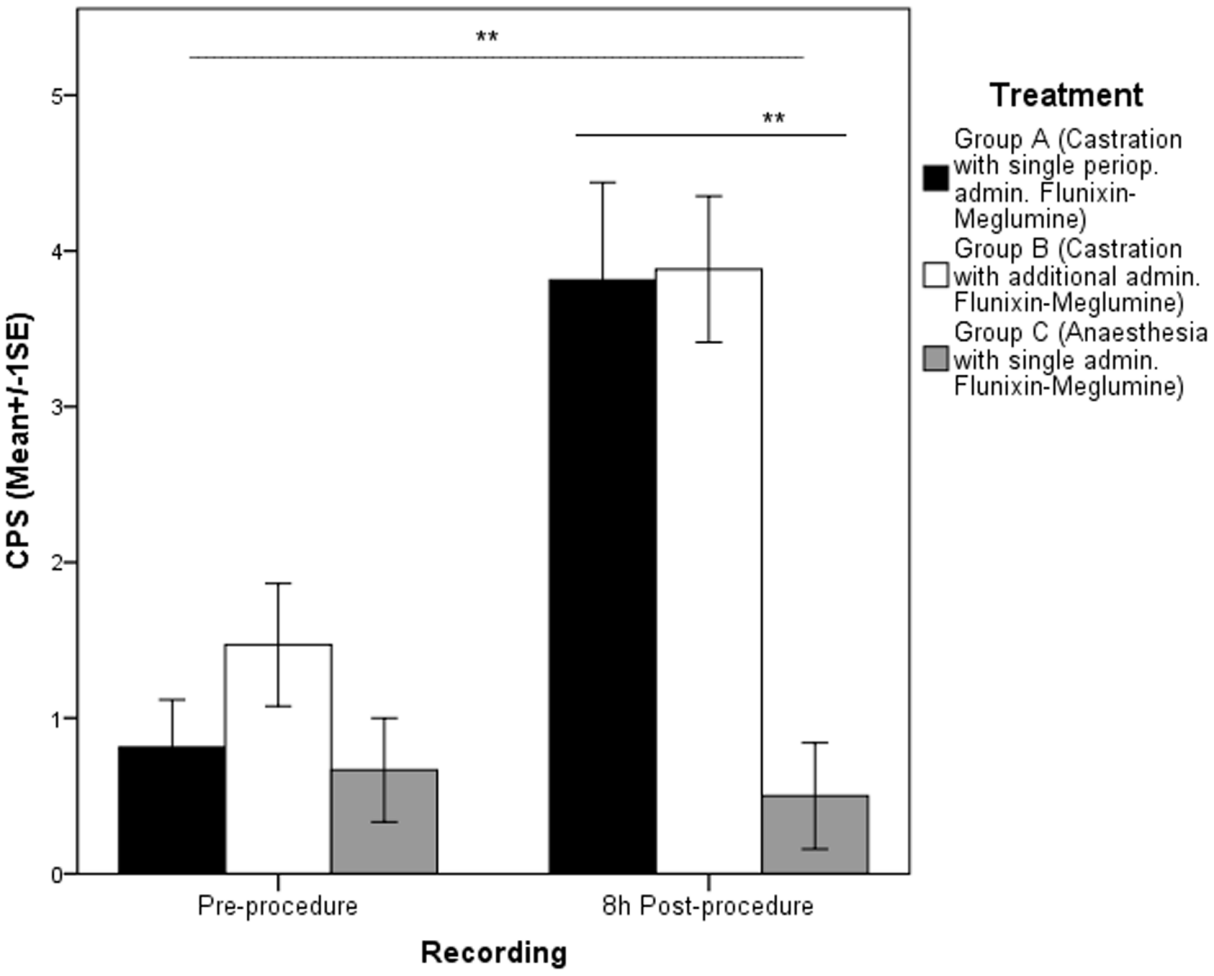
Mean Composite Pain Scale (CPS) scores pre and 8-hours post-procedure. CPS scores are presented on the y-axis (±1 SE) for horses undergoing routine castration (A and B), and anaesthesia control group (C) with the pre and 8-hours post-procedure recordings on the x-axis (** P = 0.000).

Time, treatment and time*treatment interaction had significant effects on CPS score (RGLM, P = 0.002, P = 0.002 and P = 0.050, respectively; η^2^ = 0.28). In the pre-procedure period there was no significant difference between the treatments (ANOVA, P = 0.65; η^2^ = 0.02). At eight-hours post-procedure the CPS score was significantly different between the three treatments (ANOVA, P = 0.000; η^2^ = 0.41), with the CPS score being significantly higher in horses undergoing routine castration (Groups A and B) compared to the control group (Group C) (Bonferroni post-hoc, P = 0.000 for both comparisons). No significant differences were found between groups with the single (A) or multiple (B) Flunixin administration (Bonferroni post-hoc, P = 1.000) (see Figure 5).

The CPS demonstrated good inter observer reliability between the two analgesic treatment blind observers with an overall ICC of 0.79.

### Behaviour analysis

Percentage agreement between the 2 observers was more than 80% for all the behaviours. Many of the pain related behaviours were observed too infrequently to be meaningfully analysed. Low head carriage showed a tendency to increase in duration at 8-hours after castration (Wilcoxon, P = 0.068) compared to baseline. Duration of exploration and alertness significantly decreased at 8-hours post-castration (Wilcoxon, P = 0.000 and P = 0.008, respectively) compared to baseline. The composite maintenance behaviour score (comprising the sum of the duration of exploration, alertness and grooming) significantly decreased at 8-hours post-surgery (148.1±21.7 sec) compared to pre (363.5±36.4 sec) (Wilcoxon, P = 0.000). There was no significant effect of treatment A or B on either maintenance or pain related behaviours. Total observation time needed to analyse all the videos was approximately 20 hours.

### Relationship between behaviour, CPS and HGS

The HGS score was correlated positively with the CPS score (Spearman correlation, r = 0.580, P = 0.000) and negatively with duration of explorative behaviour (Spearman correlation, r = −0.461, P = 0.002). The HGS score was negatively correlated with the composite maintenance behaviour score (Spearman correlation, r = 0.508, P = 0.001).

## Discussion

Despite the severity of pain associated with routine castration in horses being contentious [10], [38], [39], the findings of previous studies [2]–[4], [40] have demonstrated that this procedure is associated with some degree of pain. An untreated control group undergoing castration without any analgesic treatment was not included in this study for both ethical and welfare reasons, as pain can cause a long lasting welfare issue in horses [40]. Although better balanced control group would be preferable, the control group used in this study to evaluate the effect of general anaesthesia on HGS was similar (in size, age, sex, and clinical conditions) to control groups presented in other scientific studies on the assessment of pain in horses [14], [17]. As general anaesthesia for horses is not without risks for health and welfare [41], recruit more horses or healthy stallions to have a more homogenous control group would be questionable for both ethical and welfare reasons. This study has identified changes in facial expressions in horses undergoing surgical castration that appear to be similar to those previously described in other species [20]–[23], with some subtle variation due to differences in the species subjected to a variety of painful conditions. Changes in ear position, orbital tightening and some tension in the chewing muscles are largely similar to those described in other “grimace scales” [20]–[23]. In this study, differences in Horse Grimace Scale scores were observed following a routine surgical castration, with an increase in scores from pre to 8-hours post-procedure. Importantly, no differences in the HGS scores were found in control horses, undergoing general anaesthesia for non-invasive procedures, demonstrating that general anaesthesia has no effect on the HGS. Pain related behaviours and physiological parameters assessed using the Composite Pain Scale [16], [17] showed a similar pattern to that of the HGS, with only horses undergoing routine castration exhibiting differences in score between the pre and 8-hours post-surgery periods. Low mean CPS scores in relation to the maximum possible score were likely due to the fact that an analgesic treatment was administrated to all the castrated horses and that the CPS was originally developed for a broad spectrum of pain intensities (e.g. orthopaedic pain). Our results confirm the findings of other authors [4] that duration of exploration and alertness decreased in horses between pre and 8-hours post-surgical procedure. The horses showing high HGS scores also exhibited high Composite Pain Scale scores and low duration of explorative behaviour, alertness and grooming 8-hours post-surgery. Differently from other species (e.g. dogs, mice), grooming in horses was never reported to be linked to stress or suffering; whilst several authors reported that, in healthy horses, a considerable portion of the daily time budget can be consumed with grooming [42], [43]. It has been clearly demonstrated previously that pain in horses can be expressed through the exhibition of general non-specific indicators such as decrease in normal activity, lowered head carriage, fixed stare, rigid stance and reluctance to move [4], [15]. In a preliminary study on castration pain in horses, Eager and colleagues also found that grooming decreased six hours post-operatively[44]. In the present study horses undergoing routine castration showed the tendency to keep their head in a lower position 8-hours post-surgery. Although non-specific behavioural indicators of pain in equids are considered not to correlate strictly with severity of pain [15], the tendency to carry the head below the withers is of relevance because several authors reported that lower head carriage is shown in case of chronic or severe pain [18], [45]. The results of this study demonstrate that the HGS is a potentially effective method of assessing castration related pain in horses. Horse Grimace Scale scores significantly increased from pre to post castration and were unaffected by anaesthesia alone indicating that the action units relate directly to post procedure pain and/or distress. As there was no difference in the HGS between the two analgesic treatment groups, we are unable to fully differentiate between post-procedure pain and distress in this study. However, the significant difference between control and treatment groups and correlation between HGS, CPS and some non-specific behavioural indicators of pain suggest that the action units comprising the HGS are likely to change in response to pain. There are two potential explanations for lack of difference in HGS scores between those horses receiving a single pre-operative administration (Group A) and those receiving a pre and post-operative administrations (Group B) of Flunixin. It is possible that both the HGS and CPS were insufficiently sensitive to discriminate between effects of the analgesic schedules used. Alternatively, the two administrations of 1.1 mg/kg of Flunixin 6 hours apart (i.e. pre and post operatively) may not provide greater pain relief than a single pre-operative administration. Duration of pain relief of Flunixin is contradictory, Johnson et al. [46] found that additional Flunixin was needed 12,8±4,3 h after surgery, for this reason we decided to give a second dose of Flunixin before the 8-hour measurement (12,8–4,3 = 8,5 h minus time for oral absorption of Flunixin). As we did not include untreated control group undergoing castration without any analgesic treatment in this study for ethical and welfare reasons we are unable to provide insight into which explanation is correct. Therefore, further studies investigating the HGS, CPS and behavioural indicators of pain as well as the efficacy of 1.1 mg/kg of Flunixin and other analgesics with routine castration are needed to answer the above question.

The overall accuracy of the HGS (73.3%) was slightly lower than that of the other “grimace scales” (97% for the mouse grimace scale [20], 82% for the rat grimace scale [21], and 84% for the rabbit grimace scale [23]). The most likely explanation for this, is a combination of a slightly lower quality for some of the images used compared to those scored in other grimace scales and considerable variation in coat colour of the horses observed. Coat colour of the horse combined with the quality of some of the images meant that dark horses were often more difficult to score than those with lighter coats, especially if the background was dark. This issue has already been observed in mice [20], [47] where the higher the quality of the images and a contrasting background allowed the observers to more accurately score the images. Four out of six control horses had a light coat which allowed easier scoring meaning that the finding that the control horses did not present any differences in HGS before and after anaesthesia is highly reliable.

The inter observer reliability (as measured by inter-class correlation coefficients [ICC]) of the overall HGS and its component action units was similar to those of the mouse grimace scale (0.90) [20], rat grimace scale (0.90) [21] and rabbit grimace scale (0.91) [23]. As with other grimace scales applied to animals (e.g. rodents & rabbits), the observers in this study gave images of the horses in a non-painful state (e.g. pre-procedure) low but not zero scores which is inevitable when using a scale that is a composite of six individual action units. In a non-painful state these action units can be observed occasionally in isolation at a low intensity (score of 1 rather than 2), for example if an image is taken of a horse as it ‘blinks,’ then an observer may give orbital tightening a score of 1 or 2 but it is likely that they will score 0 for all the other action units. It is unlikely that HGS scores lower than two were due to stress related to being in a novel environment as all the horses were acclimated to the new environment. Using the Horse Grimace Scale to score horses ‘live’ rather than from images will help to solve this issue. The use of Horse Grimace Scale for scoring post-operative pain has distinct advantages over that of manual behaviour analysis, which can be complex due to the a greater number of behaviours that potentially need to be scored. Behaviour-based assessments appear to be more time-consuming to conduct (analysis time was 20 hours for behavioural based assessment compared to 40 minutes for the HGS). Furthermore, changes in facial expressions in the horses were detectable, without the need of approaching the subject, and by observers with differing expertise with only the HGS manual for guidance.

The HGS requires some further validation for assessing post castration pain (for instance in horse with administration of flunixin compared to horses with flunixin associated with an opioid post-surgery, considering longer follow up intervals) and could be further developed for other potentially painful procedures before it can be considered fully validated. Further studies could also be conducted to identify facial action units associated with other states such as fear and anxiety so that we are able to differentiate pain from these other states. Among the limitations of other routinely used methods of assessing pain in horses, there is considerable concern that prey species have evolved the ability to mask obvious signs of pain under specific circumstances (i.e. the presence of a predator such as humans). In humans it has been demonstrated pain related facial expressions cannot be completely suppressed by voluntary control [28] and in another prey species, for example the rabbit, it has been demonstrated that facial expressions are an easy and reliable cage-side method of assessing acute pain associated with ear tattooing in the presence of an observer [23]. It has been shown that humans tend to focus on head and face when assessing pain in humans [28] and rabbits [29] therefore this method could represent a reliable and feasible method that utilises the natural human instinct. Furthermore, HGS could be used as an animal-based indicator of spontaneously emitted pain, and it may provide insights into the experience of pain in horses in their own environment, and so be a useful tool in the assessment of horse welfare on-farm. Even though further evaluation of the HGS is required, the present results suggests that HGS may offer a reliable tool for assessing post-castration pain than other routinely used methods.

## Supporting Information

Table S1 **Composite Pain Scale (CPS) based on the one developed by Bussieres and colleagues** [16], [17] **used in this study to score pain.**
(DOCX)

Table S2 **Ethogram of horse for manual behaviour analysis.**
(DOCX)
