# Supplementary material for: Development of the Horse Grimace Scale (HGS) as a Pain Assessment Tool in Horses Undergoing Routine Castration
Source: PLoS One. 2014 Mar 19;9(3):e92281. doi: 10.1371/journal.pone.0092281 (PMC3960217; doi:10.1371/journal.pone.0092281)
Supplement: Table S1 — Composite Pain Scale (CPS) based on the one developed by Bussieres and colleagues [16], [17] used in this study to score pain. (DOCX) [file pone.0092281.s001.docx]

| **Data** | **Criteria** | **Score** |
| --- | --- | --- |
| Behaviour |  |  |
| Posture | Normal movements, stands quietly with equal weight distribution among all four legs or stand-resting with weight distribution among only three legs | 0 |
|  | Occasional weight shift, temporarily showing discharge positions, slight muscle tremors | 1 |
|  | Non-weight bearing, abnormal weight distribution | 2 |
|  | Analgesic posture (attempts to urinate), prostration, muscle tremors | 3 |
| Sweating | No obvious signs of sweat | 0 |
|  | Damp to the touch | 1 |
|  | Wet to the touch, beads of sweat are apparent over the horse’s body | 2 |
|  | Excessive sweating, beads of water running off the animal | 3 |
| Kicking at abdomen | Quietly standing, no kicking | 0 |
|  | Occasional kicking at abdomen (1–2 times/5 min) | 1 |
|  | Frequent kicking at abdomen (3–4 times/5 min) | 2 |
|  | Excessive kicking at abdomen (>5 times/5 min), intermittent attempts to lie down | 3 |
| Pawing on the ﬂoor | Quietly standing, no pawing | 0 |
|  | Occasional pawing (1–2 times/5 min) | 1 |
|  | Frequent pawing (3–4 times/5 min) | 2 |
|  | Excessive pawing (>5 times/5 min) | 3 |
| Movement | Stands relaxed or quiet movement | 0 |
|  | Reduced movement or mild agitation | 1 |
|  | Reluctance to move or moderate agitation | 2 |
|  | Refusal of movement or uncontrollable forwards movement | 3 |
| Head movement / | Natural head movements, head straight ahead for the most part | 0 |
| Notable gesture | Intermittent head movements laterally or vertically, looking at ﬂanks (1–2/5 min), lip curling (1–2/5 min) | 1 |
|  | Intermittent and rapid head movements laterally or vertically, frequent looking at ﬂank (3–4/5 min), lip curling (3–4/5 min) | 2 |
|  | Continuous head movements, excessively looking at ﬂank (>5 times/5 min), lip curling (>5 times/5 min) | 3 |
| Appetite | Eats hay readily or is not allowed to eat hay | 0 |
|  | Hesitates to eat hay | 1 |
|  | Shows little interest in hay, eats very little or takes hay in mouth but does not chew or swallow | 2 |
|  | Neither shows interest in nor eats hay | 3 |
| Auditory stimulus | Pays attention to people and noises | 0 |
| (click one’s tongue) | Exaggerated response to auditory stimulus | 1 |
|  | Excessive-to-aggressive response to auditory stimulus | 2 |
|  | Stupor, prostration, no response to auditory stimulus | 3 |
| Touch response | Contacting, no defence reaction to touch | 0 |
|  | Mild defence reaction to touch | 1 |
|  | Resistance to touch | 2 |
|  | Violent defence reaction to touch | 3 |
| Physiology |  |  |
| Heart rate | 24-44 bpm | 0 |
|  | 45-52 bpm | 1 |
|  | 53-60 bpm | 2 |
|  | > 60 bpm | 3 |
| Respiratory rate | 8-13 breaths pm | 0 |
|  | 14-16 breaths pm | 1 |
|  | 17-18 breaths pm | 2 |
|  | > 18 breaths pm | 3 |
| Digestive sounds | Normal | 0 |
|  | Decreased motility | 1 |
|  | No motility | 2 |
|  | Hypermotility | 3 |
| Rectal temperature | 36,9 –38,5 °C | 0 |
|  | 36,4–36,9 °C or 38,5–39,0 °C | 1 |
|  | 35,9–36,4 °C or 39,0 –39,5 °C | 2 |
|  | 35,4–35,9 °C or 39,5–40,0 °C | 3 |
