# Supplementary material for: Development of the Horse Grimace Scale (HGS) as a Pain Assessment Tool in Horses Undergoing Routine Castration
Source: PLoS One. 2014 Mar 19;9(3):e92281. doi: 10.1371/journal.pone.0092281 (PMC3960217; doi:10.1371/journal.pone.0092281)
Supplement: Table S2 — Ethogram of horse for manual behaviour analysis. (DOCX) [file pone.0092281.s002.docx]

| **Behaviour** | **Description** |
| --- | --- |
| Movement | |
| Stand | Standing up on all four feet or with one hind leg relaxed |
| Walk | Walking in the box |
| Trot | Trotting a few steps |
| Back up | Walking backwards |
| Not visible | Horse is not visible (or it is not possible describe what is doing) |
| Activity | |
| Alert | Paying attention to environmental stimuli (e.g. looking at, moving ears) |
| Agitation | Continuous and frantic movement (e.g. back and forth or in circle) |
| Investigative behaviour | Sniffing, licking, biting an object (e.g. box door, window) |
| Grooming | Self-grooming (e.g. rubbing and/or scratching) |
| Masturbating | Flexing its erected penis repeatedly upwards against his belly and maybe makes pelvic thrusts |
| Eating | Eating |
| Drink | Drinking |
| Urinate | Urinating |
| Defecate | Defecating |
| Vocalize | Whinny, scream and/or snort |
| Yawning | Deep, long inhalation with mouth widely opened, with jaws either directly opposed or moved from side to side |
| Licking and chewing | Pulling the tongue back and forth alternately with chewing |
| Pain-related behaviours | |
| Weight-shifting | Shifting weight from one hind leg to the other. Feet may not actually leave the ground |
| Pawing the floor | One foreleg is lifted from the ground slightly, and then extended quickly in a forward direction, followed by movement backward dragging the toe against the ground in a digging motion |
| Kicking the abdomen | Evident raising a hind leg and moving it towards the abdomen (it may reach it or not) |
| Flank watching | Turning head and neck to one flank; not always associated with touching the flank |
| Lowered head carriage | The head is held below a virtual line passing through the withers, the horse is not eating. |
| Rolling | Dropping from standing to sternal recumbency, then rotating from sternal to dorsal recumbency, tucking the legs against the body |
| Tail swishing | Quick swish of the tail |
| Flehmen | With the head and neck stretched upwards, the horse curls the upper lip back until the inside of the lip, the gums and the upper incisors are bared |
| Head orientation | |
| Window | Head is directed towards the window |
| Neighbour box | Head is directed towards the neighbour box |
| Corner | Head is directed towards the corner |
| Alley | Head is directed towards the alley |
| Not oriented | Head is not directed towards something |
| Not visible | Head is not visible |
